# Carving Up Participation: Sense-Making and Sociomorphing for Artificial Minds

**DOI:** 10.3389/fnbot.2022.815850

**Published:** 2022-06-14

**Authors:** Robin L. Zebrowski, Eli B. McGraw

**Affiliations:** ^1^Departments of Philosophy, Psychology, and Computer Science, Cognitive Science Program, Beloit College, Beloit, WI, United States; ^2^Independent Scholar, Berryville, VA, United States

**Keywords:** anthropomorphism, sociomorphing, artificial general intelligence, Participatory Sense-Making, enactivism, social cognition, social robotics

## Abstract

AI (broadly speaking) as a discipline and practice has tended to misconstrue social cognition by failing to properly appreciate the role and structure of the interaction itself. Participatory Sense-Making (PSM) offers a new level of description in understanding the potential role of (particularly robotics-based) AGI in a social interaction process. Where it falls short in distinguishing genuine living sense-makers from potentially cognitive artificial systems, sociomorphing allows for gradations in how these potential systems are defined and incorporated into asymmetrical sociality. By side-stepping problems of anthropomorphism and muddy language around it, sociomorphing offers a framework and ontology that can help researchers make finer distinctions while studying social cognition through enactive sociality, PSM. We show here how PSM and sociomorphing, taken together and reconceived for more than just social robotics, can offer a robust framework for AGI robotics-based approaches.

## Introduction

Seibt et al. (2020a) argue that social robotics/human robot interaction has a “description problem” insofar as it lacks a multidisciplinary set of terminology for describing apparently-social interactions between (at least) people and social robots. They point out that some capacities in these interactions can literally, rather than figuratively, be ascribed to robots, but that our current ontologies for making sense of these interactions fail us. Sociomorphing is the direct perception of real social capacities in agents or systems, including non-human agents. They say, “Such interactions, we proposed, should not by default and in all cases be viewed as involving a mental operation where *fictional human capacities for social interaction are imaginatively projected* onto the robot; rather, heeding a suitably wide understanding of sociality, we should allow for human social behavior towards robots to be guided by *direct (and possibly implicit) perceptions of actual non-human capacities for social interaction*” (2020, p. 63, emphasis in original). While Seibt's work here focuses on social robotics in particular, the terminology and conceptual ontology may be applicable outside of just social robotics work, and might offer important insights into artificial general intelligence (AGI)^1^ work as it relates to robotics more broadly (Seibt, 2017). We propose to tease apart the conceptual framework offered by Seibt et al. that combines sociomorphing with^1^ their larger project that then applies this new concept to an ontology of simulation. Since our project here aims at showing how this framework could help AGI work, rather than robotics work that focuses on more surface-level social interactions, we abandon Seibt et al.'s ontological commitments to simulation and imitation in favor of using these concepts in an entirely different kind of project, that of robotics-based AGI work. To this end, we include an important discussion of enactive social cognition, without which the sociomorphing work cannot get a foothold for AGI. Participatory Sense-Making (PSM) (De Jaegher and Di Paolo, 2007) offers a new level of enactive description with regard to interaction among multiple autonomous agents. As argued elsewhere (Zebrowski and McGraw, 2021) PSM is a missing and valuable variable in robotic systems as they relate to AGI (see text footnote 1). In attempting to apply PSM to robotic systems, though, it appears mysterious how and when we might attribute certain capacities, such as sentience or autonomy, to artificial systems. However, here, we propose that Seibt et al.'s notion of sociomorphing offers appropriate and useful gradations in relation to what might count as autonomous and (perhaps eventually) sentient robotic systems. In fact, these perspectival gradations offer clear theoretical distinctions that can be repurposed or reconciled with robotic approaches to AGI in ways that might capture the interesting but currently-overlooked level of description that PSM offers. Combining PSM-levels of analysis in AI work with sociomorphing helps, also, to capture the asymmetry attached to social interactions including robots, an issue hinted at but never fully explored or explained in earlier social cognition work (De Jaegher et al., 2010)^2^. Additionally, while our goal here is to present a synthesis of these two approaches and show how they specifically can work in service of the AGI project that neither is individually aimed at, we also recognize that targeted experimentation within this framework, like all frameworks, is the next step to making sense of this problem and measuring the degree to which this new conceptual toolbox produces fruitful results. Therefore, our task here is to iteratively explore these concepts, and evaluate the terminological innovation as a sort of act of conceptual engineering in service of making sense of sense-making in concert with non-humans.

We recognize that the structure of this paper is a bit non-standard. We believe that this is necessary in order to incorporate the jargon of multiple niche academic areas in a way that will be understandable by naïve readers. To be clear, we understand our contributions to the literature to be as follows:

1) We borrow the concept of PSM from enactive social cognition and show how it is a valuable framework for making sense of behavior between humans and robots, while recognizing that this was not the domain it was designed for.
2) We borrow the concept of Sociomorphing from human-robot interaction (HRI) and show how it can be applied beyond the social robotics it was designed for, enlarging the scope of robotics-based AGI projects to help avoid known problems with anthropomorphization.
3) We offer a new conceptual framework for robotics-based AGI projects with the goal of focusing on social interaction as a methodology and location to study primary cognition with either or both human/animal and/or robot agents.

As such, we implore the reader to stick with us as we attempt to make various vernacular jargons familiar enough to work with, and hopefully to further deploy in AGI research.

The general structure of the paper is as follows: in Section The Argument, we offer the argument as we understand it, explaining PSM and its potential role in AGI, along with empirical support for the claims, in Section Background: PSM. In Section Sociomorphing, we discuss what sociomorphing is and why the concept is needed, both in HRI as it was originally intended, and also in AGI as we are applying it. In Section Background: Concepts of Sociality, we discuss the ways non-humans have been conceived of in social interactions across a range of literatures from various disciplines. In Section New Language and Conceptual Engineering, we offer evidence that anthropomorphism has failed to properly fulfill the explanatory role for which it is intended. In Section Failures of Anthropomorphism, we begin to put the pieces together to combine PSM and sociomorphing in a more complex way. This leads us to Section Revising “Social Interaction With Robots” in which we solidify the nuances of sociomorphing as a process, and try to show how its use forces a revision of our understanding of social interaction with robots, with a focus on perspective-taking. In Section Phenomenology, we enrich this argument by showing how it has explanatory power to make sense of the phenomenological experiences described by various human interactions with non-humans. Then in Section Autonomy is Hard, we revisit earlier complications involving senses of autonomy that need revision to reconcile enactive social cognition with robotics. We close the paper with a discussion in Section Discussion.

## The Argument

### Background: PSM

In the classic example, we are asked to imagine two people trying to pass in a hallway, and frustratingly mirroring one another instead of successfully fulfilling the intentions of each, which are to simply keep walking. As opposed to traditional cognitive approaches to social cognition, in which an interaction with another person tends to be just a special instance of in-the-head cognition (in the form of mindreading or simulation), within enactivism, social cognition is a richer and more fundamental kind of cognition, in which genuine new kinds of meaning-making are enabled. In articulating the enactive concept of social cognition, De Jaegher and Di Paolo (2007, 2008) name it “participatory sense-making” (PSM). With roots in biological autonomy, the most general claim is that some kinds of social interactions produce a kind of cognition/sense-making that emerges in the dynamics of the interaction and cannot be reduced back to the intentions of the individual actors.

Cognizers have a consistent perspective of their world because of the precariousness attached to their self-organization and self-maintenance, which entails needs and constraints relevant at multiple levels of their identities. Think of it this way: I need food, and I also need a friend. “A social interaction is an autonomous self-sustaining network of processes in the space of relations between the participants, provided their autonomies are also sustained” (De Jaegher et al., 2018, 139). An interaction process, in particular one that's considered autonomous in the right way, has patterns of coordination and breakdown which parallel the needs and constraints relevant to an individual sense-maker. Meaning, also, emerges in the same way from those patterns of coordination and breakdown which necessarily incorporate and sometimes supersede the processes surrounding the two (or more) individual sense-makers involved. Not all interactions count as social, and those that do have self-maintaining tendencies. In other words, the people involved in the interaction coordinate part of the exchange, and the exchange itself feeds back and encourages them to further sustain or modify the interaction. Think again about the people trying to pass in the hallway.

Recently, it has been argued that AI and AGI work has failed to consider and include claims from enactivism broadly and PSM specifically (Zebrowski and McGraw, 2021). In rethinking autonomy and openness in light of the enactive framework, social cognition, especially in this form, is highlighted as a central faculty of AI that has been widely overlooked. PSM has opened up a new research program to pursue with respect to AGI (and social robotics work). Yet what constitutes a sense-maker, and what is needed to produce an interaction process which fits the criteria necessary to be considered an *autonomous* sense-making thing in itself remain unclear in some conditions, largely as a result of both the traditional problem of other minds and also the empirical facts about human social behavior. In other words, there remain problems in describing and determining what constitutes a genuine sense-making system, particularly in regard to robotics, without running into epistemological roadblocks complicated by human tendencies toward anthropomorphising, and conflicting ideas of autonomy itself between enactivism and robotics (e.g., Haselager, 2005). In some of this recent work it is pointed out that there is a longstanding lack of consensus around concepts of agency and autonomy in the robotics and AI communities, as well as within many different systems of philosophical analysis (Zebrowski and McGraw, 2021). Drawing on Haselager (2005) and Barandiaran and Moreno (2006) the authors say: “In the most uncomplicated sense, a system is understood within robotics to be autonomous when it is able to perform its work without oversight. A robot is generally understood to be autonomous in the relevant sense when it acts in a way that precludes any human from being in the loop, and (perhaps more controversially) when it does so in an unpredictable environment. But a human is often considered autonomous in a much more radical sense: human autonomy tends to point toward a kind of metaphysical claim of free will… The least controversial sense of human autonomy is one that is limited to the ability to set and pursue one's own goals (309). They go on to say, “Within the enactive and PSM literature, an autonomous system is simply a system under precarious circumstances whose processes work to generate and sustain many of those processes as a source of self-identity” (310). So while the question of autonomy is a messy one, especially considering different fields and domains having distinct language and concepts, these ideas are not irreconcilable. The most convincing reconciliation comes from Di Paolo (2003), when he asks, “how can we invest artfacts with a similar sense of meaning? Do we need to go all the way down to metabolism and self-production or is another solution possible?” (12). He argues, instead of relying on the prototypical case of enactive autonomy, that of processes of life, we can focus on “the mechanisms for acquiring a *way of life*, that is, with habits” (13). Imbuing artificial systems with something like Deweyan habits then becomes an example of a way forward in making sense of autonomy that isn't limited to living systems. We will return at length to discuss anthropomorphism as it relates to this question, since this is the part of the equation that can be dealt with empirically.

Importantly for our purposes, we want to emphasize that enactivism has a complicated history with AGI. It is often ignored for its starting point in biological autonomy (although taken up and overlapping in some ways with historical uses of cybernetics). However, thinking about social cognition at all, and enactive social cognition in particular, would be an invaluable addition to AGI projects. Because of the historical bias in which minds are thought of as private, internal structures, social cognition tends to be an afterthought, if it is thought of at all in AGI. What PSM offers us is not merely the internal flipped outward, but a recognition that the private internal mind was never the right starting point. Instead, as PSM shows us, our interactions with other agents, as well as with the world, are the right starting point for making sense of social, and even individual cognition.

Reconciling an enactive theory of social cognition with a range of AGI projects, however, is difficult in multiple ways. One notable way is that PSM carries with it some assumptions about the kinds of systems that can be meaning-makers within these interactions. While teasing this apart is a large part of our overall project in this paper, we want to highlight one particular issue that deserves notice, because it is under-explored in the literature. Because only certain kinds of (usually biological and social) creatures can be interactors in obvious ways within PSM, there is a kind of assumed symmetry within these interactions. If we are engaged in joint meaning-making, we must both be social and cognitive creatures in at least roughly the same way. This symmetry proves particularly tricky in working out human interactions with animals, as well as with non-biological systems like social robots or other artificial cognizers (should such a thing 1 day exist).

There is empirical support for many of the claims in PSM across multiple methodologies and levels of description, from modeling at the neural level all the way to full embodied action (Reed et al., 2006; Auvray et al., 2009; Candadai et al., 2019). For example, Reed et al. (2006) performed an experiment in which two people might be linked haptically in trying to solve a target acquisition task. In the experimental condition, the two people were linked, and responded to one another's motor control systems in trying to acquire the target, although they were ignorant of whether they were in the experimental condition or not. Subjects either independently moved a handle to place a projected mark into the target (in the single condition) or were tethered together in solving the problem (in the dyad condition). The results are surprising, and a reminder of why group or collaborative work, while often frustrating, tends to produce better outcomes than working alone. The authors say, “…task completion times indicated that dyads performed significantly faster than individuals, even though dyad members exerted large task-irrelevant forces in opposition to one another, and despite many participants' perceptions that their partner was an impediment” (365). In spite of feeling frustrated by their partners' real and perceived hindrances, pairs were faster and more successful at the task. Individual intentionality cannot aim toward this end; it can only be reached through an interaction with another person [see also a discussion of this experiment in De Jaegher and Di Paolo (2008), 143–144]. Similarly, Auvray et al. (2009) designed an experiment using a one dimensional plane on a computer screen to show that human participants can consistently detect and distinguish the presence of another person from that of both a fixed object and one that is mobile, as well as one that's a lure, shadowing the other participant's actual position. Otherwise sensory-deprived participants were given haptic feedback when crossing one another's activity, and the same feedback when crossing a fixed or mobile object. Each participant was told to perform an action (click a mouse) when they believe they've encountered another living participant. The authors state, “When the trajectories of the avatars cross, *both* participants receive a stimulation… each participant then turns back, then they will meet again, and this pattern forms a relatively stable dynamic attractor” (11). Given the sensory motor dynamics of the interaction, and the patterns of activity which arise through active engagement with the other living participant, they tended to create “…joint strategies of mutual exploration” resulting in the participants finding each others' avatars more often than not. These studies suggest that when two sense-making systems interact with one another (in regard to completing a specific task), an interesting new level of description tends to emerge between those two agents, one that couldn't have emerged for just a single individual. Thus, we see PSM in action: at least two autonomous systems in interaction, producing a new autonomous system that is dynamic and responsive to the individual interactors, but not always in a predictable or desirable way. It is also suggested that dynamical systems tools can model and measure this system.

### Sociomorphing

Bracketing for a moment the phenomenological experience, we want to acknowledge that there are many interactions between humans and robots, as well as mundane interactions between humans and animals, that seem to involve genuine meaning-making. Due to limitations on the kinds of robotic systems we have at this point in history, the meaning-making is largely one-sided in those interactions, but the system in interaction may well be autonomous enough that it will soon, if it doesn't already, count as its own rudimentary kind of cognizing system in a PSM-style interaction. But this asymmetry in interaction requires serious attention, especially if we ever hope to make the leap away from biological autonomy as the only actual (or conceptual) possibility of meaning-making.

In recognizing a conceptual and terminological gap in HRI research, Seibt et al. (2020a) have argued that the persistent approach of analyzing robots through the lens of anthropomorphism is mistaken; in its place, they offer a new ontology that takes account of perspectives (both of participants and observers) with a focus on asymmetrical social interactions. They argue that anthropomorphism as a frame hinders our ability to make sense of and study human interactions with social robots (in particular) because we mistakenly believe we impose human capacities and characteristics on machines which do not have them. Instead, they argue that there are genuine social capacities in animal and robot systems, but that we do not yet have a framework for understanding those social capacities in any way other than imposing human capacities on them. We are always already aware of the non-human capacities in some of our social interactions, and we already make adjustments to our behaviors based on that awareness, which isn't fully captured by an anthropomorphic analysis (“e.g., one can undress in front of a dog without being ashamed”) (Seibt et al., 2020a, 63). In other words, you don't treat the dog as a person with human social skills and capacities, but you automatically make different judgements about its role in your social world. This is likely also true of, say, a robot dog like Aibo, although not necessarily in the same ways that it's true of a biological dog. Rather than anthropomorphizing, this, then, is sociomorphing.

What sociomorphing adds to this picture is a way to sidestep some of the problems of enactive autonomy, by offering a new conceptual framework in which we can conceive of asymmetrically-distributed social capacities across different kinds of systems. One of us (Robin) has a dog who appears to engage in frustratingly social interactions, wherein she (the dog) will steal an object that has been placed deliberately but unsuccessfully out of her reach, and then bring it into view so that whoever is nearby will attempt to retrieve it from her. This usually looks like Robin chasing the dog around angrily and yelling futilely, the dog appearing to greatly enjoy this interaction, running more the angrier Robin gets. This would appear to count as a kind of PSM, insofar as there are two autonomous beings engaged together in a single act, both of whom appear to be frustrating the intentions of the other, but both of whom also continue to engage not despite, but because of that frustration. Robin wants to get the object without chasing the dog, and the dog wants to be chased and sees the object only of instrumental value in reaching that goal, and yet both are drawn into this game repeatedly. You can see how the language of intentionality, when projected onto the dog, is controversial and less than ideal. While the game feels and looks quite deliberate from the human side, it is extremely difficult to project intentions onto a dog like this without running afoul of so much work in cognitive ethology. What sociomorphing offers is a new category of explanation, a new set of tools and language embedded in a whole new framework, by which we are already engaged in treating the other with respect to its actual capacities and not imagined human capacities we know it doesn't possess. We anticipate different kinds of responses from a dog, or a robot, than we would from another person, and we react in the situation to those *actual* social capacities, not as if the other is capable of the narrow kinds of human interaction that anthropomorphism seems to demand.

### Background: Concepts of Sociality

While PSM offers a valuable lens through which to examine social interactions, particularly those involving humanoid robots or potential future AI systems, there have long been questions about the role of non-humans in such interactions. In examining the concepts of social interaction and social cognition, De Jaegher et al. (2010) introduce the possibility of robots being genuine social interactors under the right conditions. To reiterate, they say, “We do not restrict social interaction to the human species. As long as the terms of the definition can be verified, they can apply to cross-species interactions or interactions with robots that are autonomous in the sense intended” (443). We are left wondering how autonomy “in the sense intended” can happen in non-living systems, given that the sense intended generally centers processes of life and autopoiesis. The authors gesture toward future research questions, many of which overlap with our own here, including explorations of the characteristics of asymmetric social interactions and observational social understanding (such as watching a movie) (446).

There is no interdisciplinary consensus on the role of non-humans in social interaction, although reviews of the literature have been undertaken in several disciplines with stakes in the answer. Cerulo (2009) offers a broad review of theories mainly in sociology, but with reach into philosophy and psychology, too, which considers ways in which various forms of non-humans might fit into social interaction. A theme that emerges in Cerulo's review is that non-humans have been considered potential social interactors across a wide variety of theories, playing different roles and having different constraints. Most importantly, what emerges from this literature review is that interaction processes as well as the entities that potentially contribute to them have consistently gone through revision in tandem with evolving theories and technologies. For example, both Nass and Turkle noted that we interact with certain technologies and objects in fundamentally social ways,and in some cases those objects are capable of actively evoking feelings of intersubjectivity in us as we interact with them (Cerulo 539–540). Owens and Cohen, on the other hand, suggested that we consider non-humans “doing mind” in social interaction, understanding the non-human interactant as an other, treating it as independent of oneself and acting as if it has the capacities it seems to have (536). Importantly, this is not just projection and anthropomorphism, at least according to these theorists, indicating that this debate predates questions about AGI and social cognition.

In large part, Cerulo suggests that nonhumans in general “…deserve a more central place in our analytic frame” (543). She posits that the role and function of the mind in social interaction has been lacking, and that shifting focus to a more inclusive frame will help to fold in “…entities capable of different states of mind” (543). She claims a dog, for example, has been shown to have some awareness not only of “interactive routines” but also to establish “…cognitive, affective, and behavioral presence in interaction…” (Cerulo 544). This seems undeniably true in the case of Robin and her dog, but what of someone and their Aibo? Where the traditional (mostly sociological) theories Cerulo puts forward in her literature review have attempted to incorporate (in some form) nonhumans in interaction, they've not been definitive, in particular because social robotics and humanoid robots pose new kinds of problems, many of which relate to and are exasperated by failures of anthropomorphism.

Of course, philosophers of technology and others in human-robot interaction have long tackled this same question of sociality, asking what role the robot does or can play in our social interactions. Mark Coeckelbergh, in a paper laying out a new way to approach human-robot relations, discusses technology as an “other in itself.” This work shifts the conversation away from what an entity *is* to what it *appears to be* (Coeckelbergh, 2011). The author uses Idhe's concept of an alterity relation to strip away the idea that an entity must have some prerequisite form of intentionality or consciousness to be considered properly social in human-technology relations. He sidesteps the ontological unknowns by focusing on “the appearance of the robot as experienced by the human” (199). Although Coeckelbergh's framing is phenomenological rather than sociological, like Cerulo's, the idea here links to an overarching conversation touching on anthropomorphism's cross-disciplinary relevance, and the mistakes therein. There is a large body of scholarly literature in both social robotics and social cognition that attempts to tackle these questions, but because there remains no cohesive HRI field that captures each of the relevant disciplines, the problem remains (Hakli and Seibt, 2017). A centralized, cross-disciplinary framework is necessary to at once sync fractured theories together and also to move toward a more cohesive tool in understanding the complexity surrounding social interactions, in particular asymmetrical ones. For our argument, this is a prerequisite to us being able to use this new combined framework and conceptual scheme described here.

The scholarly literature on non-humans in sociality shows the limits of anthropomorphism in making sense of social interaction broadly. Even the phenomenologically based relational views put forward by Coeckelbergh and others focus heavily on a human's experience of a social robot through an analysis shaped in terms of anthropomorphism. To this end, Seibt et al. introduce the concept of sociomorphing, which is the perception of real social capacities and characteristics, but not framed in terms of human sociality, which we have taken up here.

## New Language and Conceptual Engineering

### Failures of Anthropomorphism

As mentioned, it has been argued that the field of AGI could benefit from the conceptual approach offered in PSM (Zebrowski and McGraw, 2021). Briefly, this is true because the enactive approach to social cognition offers new potential levels of cognitive analysis through the form of emerging dynamic systems in social interaction. This joins historical calls to center 4e cognition and embodiment, including humanoid embodiment, in AGI (Holland, 2007; Chella et al., 2019). Zebrowski and McGraw (2021), which we build on here, centers on reconciling enactivism's biologically-based notion of autonomy with muddier notions of autonomy used across robotics and AGI, as mentioned in Section Background: PSM. Using the conceptual and empirical tools offered by PSM, progress might be made toward understanding the possibilities enabled by certain kinds of artificial systems in certain kinds of social interactions with certain kinds of living systems. However, this picture leaves open the possibility of perceptually-indistinguishable but ontologically-distinct pictures, like the traditional philosophical zombie problem.

If PSM is going to be a valuable framework for understanding social robotics and (more importantly for our purposes) future AGI work, then we need to understand the role of the non-human system in that interaction better than any framework currently does. In spite of an exploding literature in human-robot interaction (HRI) and social robotics as they relate to anthropomorphising, Seibt et al. newly suggest that anthropomorphism isn't actually all we're doing when we as humans engage with (social) robots. In other words, part of the roadblock in making sense of social interactions with robots broadly, and within enactive frameworks specifically, has been a lack of terminology to make proper sense of the ontological status of each participant. Sociomorphing, then, is a “terminological innovation” that provides us with conceptual and empirical approaches not previously accessible within this framework.

The traditional accounts of social interaction imply or explicitly demand that all interactants have some number of certain kinds of (human) social capacities, including consciousness, intentionality, self-awareness, empathy, emotions, beliefs, reasoning, capacity for joint-action, etc. (Duffy, 2003; Cerulo, 2009; Hakli, 2014; Parviainen et al., 2019; Damholdt et al., 2020; Seibt et al., 2020a). With regard to human-robot interaction (often social robotics), the literature on anthropomorphism has always been contentious (Duffy, 2003; Waytz et al., 2010; Darling, 2017; Epley, 2018; Zebrowski, 2020). Many researchers point out that our projection of human capacities onto non-human systems results in a *metaphorical* use of anthropomorphism already. Parvianen et al., for example, say “currently, the robot functions are described metaphorically in the human-robot interaction literature, which refers to human consciousness capabilities. Robots are said to “sense,” think,” and “act” (Parviainen et al., 2019, 323). Duffy (2003) points out that anthropomorphism “includes metaphorically ascribing human-like qualities to a system based on one's interpretation of its actions… [it] is a metaphor rather than an explanation of a system's behavior” (181). Within social robotics in particular, where the systems are designed to look social and appeal to evolutionary responses people may have that judge certain traits as social, we can easily see the mismatch between the concept of anthropomorphism and its application.

One way that anthropomorphism seems to fail as a proper frame in human-robot interactions is that there is a wide gulf between the kinds of human capacities that produce certain behaviors in humans and those similar kinds of behaviors in robots (linguistic behavior here is the most obvious; when I say “I love you” it means something very different than when an Aibo or a Pepper robot says it. The same is true of many other imitative behaviors). There is an asymmetry in these interactions that causes a shift in perspective as to how I understand the robot's actions, and how I understand what the robot will understand of my actions. This asymmetry isn't new, and it holds for many of our human-animal interactions, as well as interactions with humans who differ from us widely in age, class, or neurotypicality^3^.

There has long been a call for new language both to conceptualize and study human interactions with robots without relying on anthropomorphism. Duffy (2003) paper includes a clear recommendation for this: “Anthropomorphism should not be seen as the ‘solution' to all human-machine interaction problems but rather it needs to be researched more to provide the ‘language' of interaction between man and machine” (181). Coeckelbergh (2021), also calls for an overhaul of our understanding of anthropomorphism as it relates to social robots. In writing about the use of social robotics in relation to social interaction, Hakli (2014) gestures to the shortcomings of sociality broadly, anthropomorphic tendencies more specifically, and the need to produce new language which takes into account the breadth of the social. He surveys various ways social robots have been excluded from the concept of sociality by definition, ruling them out by defining social interactions in terms of consciousness or intentionality. Instead, he argues that perhaps “if people conceive of their interaction with robots as social interaction, this should count as *prima facie* evidence that their interaction with robots is social interaction” (107). Conceptualizing sociality as fluid and malleable rather than having well-defined boundaries can completely alter our ways of understanding social cognition. These definitional pitfalls, to Hakli, shed light on the need to rethink the concepts that provide structure for our theoretical approaches in HRI and with asymmetric interactions more broadly. Hakli points to the fact that other theorists have undertaken the challenge to “…build conceptual frameworks with graded notions and several dimensions that enable us to locate more carefully the differences between different types of agents” (113). Seibt et al.'s project with regard to sociomorphing is an attempt to answer these calls for new language and concepts, and we hope to take this challenge up and apply it further into AGI work, too.

### Revising Social Interaction With Robots

On some level, we're asking how we can judge that a robot has developed a mind, but this framing misses all of the nuance needed when trying to make sense of something long misunderstood as a binary. While there's a simple way that this is nothing more than an illustration of the traditional problem of other minds, it seems that taking up (Seibt et al., 2020b) framework helps chisel away at more of the problem through clarifying and reconceptualizing some of the framework we superimpose in analyzing human-robot social interactions. In other words, what has the luxury of being a purely theoretical issue for philosophers is much more pressing for practitioners, and we hope to concretize some of this theory for researchers to take up in practice. In approaching these interactions as involving sociomorphing instead (or alongside) of anthropomorphizing, we shift our focus to the actual capacities of the artificial system instead of fictionalizing the interaction as if it were between just human interactors. When I interact with another person, I know that the social capacities for our interactions are more or less symmetrically distributed, and that guides the perspectives I take on such an interaction. But when we interact with some kinds of social robots or animals, the authors argue that part of what we do is generate a new model that tries to account for the asymmetry of the interaction. Sociomorphing involves all of the following (p. 58):

“(S1) it is *direct perception*;
(S2) it is a perception of *non-human* characteristics and capacities (which resemble certain characteristics and capacities familiar from human social interaction to different and possibly very low degrees) in non-human entities and circumstances;
(S3) it both arises in and guides *interactive sense-making* in a situation of practical interaction (or the perception of an interaction);
(S4) it typically occurs preconsciously but may also occur consciously; and
(S5) it pertains to (relative to an external observer) *actual* features of non-human entities and characteristics.”

**Figure 1:**
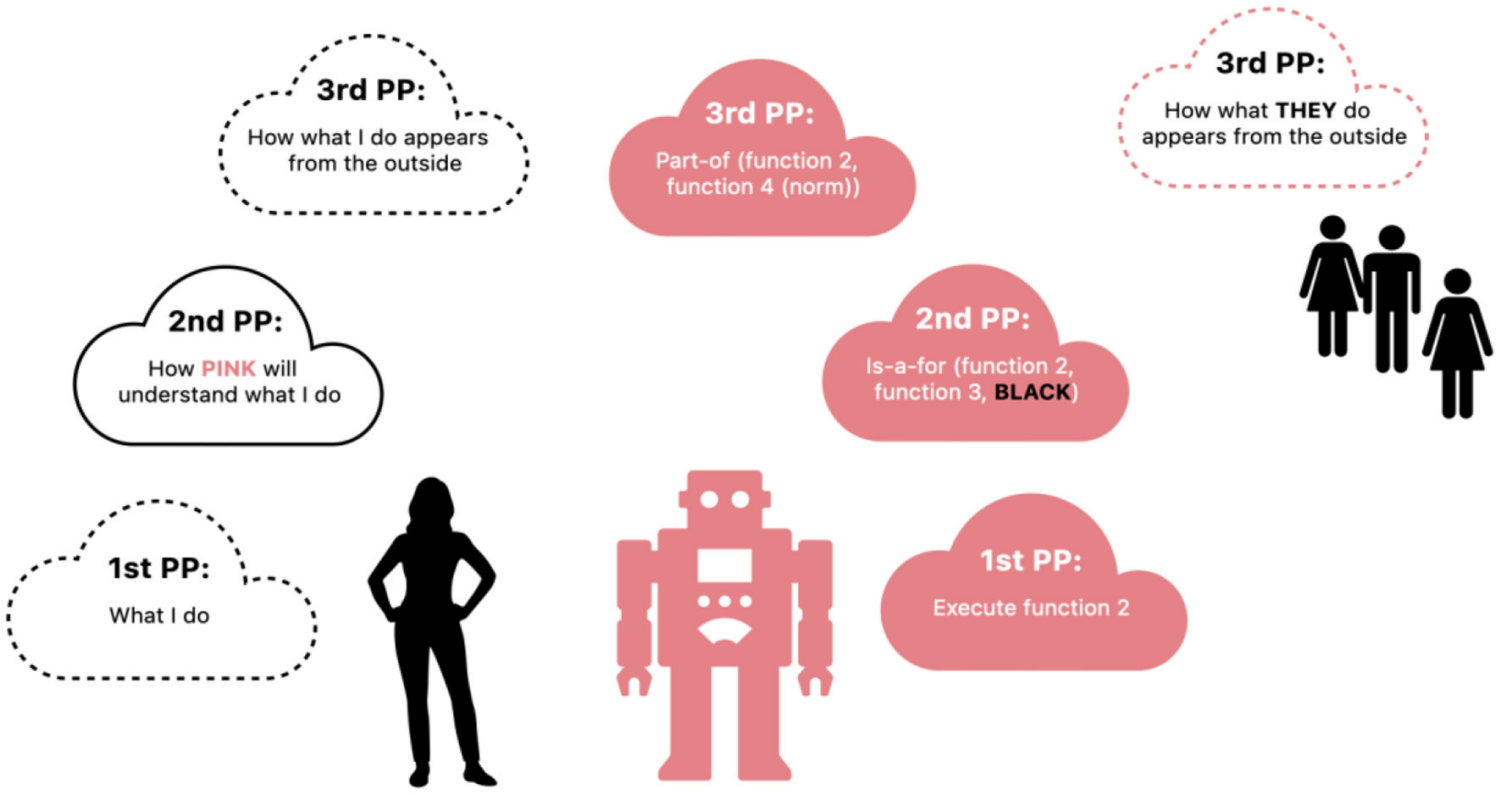
The modified second-person perspective in asymmetrical social interactions, adapted by Eli McGraw and Jacqueline McGraw from Seibt, Vestergaard and Damholdt 2020, with permission.

We implicitly pick up on the shifting perspectives needed to move from symmetrical to asymmetrical social interactions, largely shifting the second-person perspective (see Figure 1). If we look at this figure, we can see the seven perspectival perceptions of an asymmetrical social interaction with a social robot as described by Seibt et al. In a default social interaction, for example between two peers, they sociomorph one another using that default second-person perspective with the assumption that “the capacities required for the present kind of social interactions are symmetrically distributed” (61). However, in this figure, we can see how the human, possibly implicitly, understands that the robot will take the human's actions differently than a peer would, and changes the interaction accordingly. My understanding of my own action is different when I consider (through the second person) how my actions are being perceived by a robot or a dog (for example). There would be a cascade of changes given the “non-default” ness of an asymmetrical situation. While the authors here point out seven specific perspectives involved in this sort of interaction, elsewhere they write that social interactions have “irreducible perspectival complexity” (Seibt, 2018, 140; Seibt et al., 2020a, 137). We suspect that the designers of the robot also need to be represented in this picture, as the human interacting with it will tend to default to what they think the system was designed to do, but detailing the richness of these perspectives is beyond the scope of this paper^4^.

### Phenomenology

Recall that the primary aim of sociomorphing is to recognize the perception of actual social capacities in a thing or person without assuming evenly-distributed symmetrical social skills. Along with this, the authors introduce a concept of types of experienced sociality (TES) understood as the feelings of co-presence with another agent or entity. They hypothesize that “sociomorphing can take many forms each of which is manifested in, or otherwise associated with, a *type of experienced sociality*” (Seibt et al., 2020a, 52) (emphasis in original). New TES's occur when one agent “operates with a 2nd person perspective that deviates from the symmetry assumption” (61). A portion of any given TES touches on a “feeling of co-presence or ‘being-with”' a particular social interactor (59). Imagine, for example, the phenomenological distinction between what it feels like to be in a shared space with a dog, or cat, vs. with a Pepper robot or another human being. They acknowledge that in some cases, these capacities may be perceptually indistinguishable from corresponding human capacities (making this an ontological claim, but one that can be tested or deployed in empirical settings to make finer distinctions within the research). The feeling of co-presence is relevant in each case, but the type of experienced sociality changes given differing expectations of the situation, including things like anticipated responsive capabilities and environmental circumstances. Some forms of sociomorphing and the TES's associated with them can be mapped onto others intuitively. The TES of being-with PARO or an Aibo for example, might resemble that of being-with a cat or dog, or it may not at all, depending on the context of the interaction. These complexities which arise within two conceptually similar TES's point to the idea that a new descriptive framework is needed to better anchor various forms of sociomorphing, and the shifting asymmetries across different (actual and potential) sense-making systems.

Including this phenomenological feature, the TES, in the ontological picture is long overdue. The experience of being-with certain kinds of animal-like or human-like robots has long been reported as similar to being-with a being. For example, Turkle (1995) reported her first experience with MIT's Cog as having been surprising, since she knew what the robot was and what it was (not) capable of. But still, she says, “Cog ‘noticed' me soon after I entered its room. Its head turned to follow me and I was embarrassed to note that this made me happy. I found myself competing with another visitor for its attention… Despite myself and despite my continuing skepticism about this research project, I had behaved as though in the presence of another being” (266). Similarly, Darling (2021) reports a surprising experience when visiting Boston Dynamics. She, like Turkle, had plenty of experience with the generally non-functioning robots in the labs at MIT, and when she saw one non-functioning robot slumped over at Boston Dynamics, she remarked that people often think the robots are more functional than they ever actually are. But she goes on, “My jaw dropped. Behind the door was a gymnasium-sized hall outfitted with an elaborate obstacle course. Dozens of dog-sized robots were roaming the premises, walking up and down stairs, pacing back and forth in pens, or ambling around the area completely by themselves” (Darling, 2021, 102). In spite of their rich familiarity with robots, each of these researchers reports a surprising TES, a phenomenal experience of being-with another kind of living being. This phenomenon is not new and has been widely explored in relation to phenomenology (Zebrowski, 2010). Darling's language clearly draws on the experience of being-with dogs, but it remains unclear how much she sociomorphs the Boston Dynamics robots as dogs and how much she instead implicitly takes up a similarly non-default robotics-based perspective in this interaction. For this reason, assumptions and intuitions about the TES and associated variety of sociomorphing cannot be determined in advance of targeted research within this framework. Given this, both empirical and theoretical researchers in HRI and AI ought to take up the sociomorphing framework to better understand how people without as much experience with robots as, for example, Turkle and Darling do, will experience them as animals or people, and what role these systems can play in social sense-making.

Our TES associated with being-with a dog or baby is likely repurposed with a social robot until the robot speaks (like Sony's Aibo); imagine encountering the fictional Lying Cat from the comic *Saga* (Vaughn and Staples, 2012). (Lying Cat is a large bluish cat in the comic, who speaks one word, “lying” when someone is lying. His metaphysical capacity to always know this is never explained, and reflects no counterpart in the actual world we live in). He, too, is a kind of categorical novelty in the same way as the social robot, and sociomorphing captures the experience in a way anthropomorphizing does not. The capacity of a large cat to be a sense-maker in an interaction process depends in part upon the way it fits my preconceived idea of what social action looks like through my own lived experience. Importantly, though, I am already sociomorphing and considering a new TES that goes beyond the scope of any previous interactions in such a way that introduces the perception of the manifestation of a new capacity (because cats have never done this). The joint process that arises between myself and Lying Cat then, can be considered in terms of a new *something* not previously available explicitly within the enactive social model of PSM. Therefore, the sociomorphing framework enriches our application of PSM to AGI specifically, and not just HRI as proposed by the authors. We again suggest that this enlargement of this framework has explanatory power beyond interaction between humans and simulation-based systems.

## Autonomy is Hard

We began this paper claiming that PSM is an overlooked theoretical approach to social cognition that researchers in AGI would be wise to take up. This is the case because it describes the social interaction in terms of autonomous systems, which, in proper kinds of interaction, generate new autonomous systems capable of being studied on their own. It denies the “rear-window” approach to social cognition, refusing to allow in-the-head intentionality to be the appropriate level of description as it remains in both mindreading and simulation theories. It explains empirical data that shows how multiple people in interaction perform in ways that are more than the sum of their parts, as well as offering a richer, enactive view of cognition that doesn't merely apply individual cognition to two or more people.

PSM, though, is a system requiring multiple levels of autonomy: the individual interactors as well as the emergent dynamics under the right conditions. At the core of these autonomies and interactions is what Di Paolo et al. call a “primordial tension.” This is not a tension between two agents in interactions, but between the agents and the situation in which PSM emerges. This tension persists “even if others are not present *as others* or if there is no discord between intentions at all” (140). In other words, even if you aren't aware that some of your interactions are with another cognitive system, PSM may still occur. While we've avoided saying too much about autonomy itself here, we must point out that autonomy in the enactive sense is based in life processes, or at least a kind of self-sustaining system which has not yet been achieved in artificial systems. Autonomous robots, as the term is used, share very little with autonomous systems within the enactive framework, and we want to be careful not to equivocate to solve the problem. However, as mentioned in Section Background: PSM, Di Paolo (2003) has posed a potential way to resolve the conflict by refocusing enactive autonomy away from life processes and towards a “*way of life”* (13), a difference that can make a difference in actual robotics-based AGI research. Seibt et al.'s framework of sociomorphing and types of experienced sociality (TES) help us reconceive the interaction process in a way that opens up possibilities of gradations in how we understand autonomy. Instead, the interaction is understood as a new kind of thing, that requires new perspective taking. This reframing allows for new ways of thinking about interacting with agents that may or may not (yet?) be autonomous in the sense intended.

For example, in the Reed et al. (2006) study discussed earlier, two people are interacting to solve a task, in spite of being frustrated by the interaction process (indeed, people were unaware of which condition they were in, the single condition or the dyadic condition, and hence were unaware of the other *as other in interaction)*. The interaction, in this case, enables the cognition needed to solve the problem quicker and more accurately than either could solve it alone. The interaction process, in this case, seems to, “deliver the necessary cognitive performance” (De Jaegher et al., 2010). In other words, social cognition is not just enabled here, but constituted in and by the interaction process itself, as PSM predicts. As summed up by De Jaegher et al. (2018) “in cases of synergy between individual and interactive normativity, acts acquire a magic power. They achieve more than I intend to” (143). We are arguing that this magic power may actually emerge among other systems of sociality, including possible or actual emergent dynamics between human and artificial systems.

Take Sony's Aibo robot dog, for example. In 2015, the New York Times documented the phasing-out of Aibo, and the impact it was having on multiple Japanese families who were holding funerals as the Aibos became unrepairable. Aibo was a categorical novelty insofar as it was a robot, but it looked (sort of) like a dog, and it was trainable, so each person's interactions with their Aibo would change the system to optimize future interactions with that particular owner or family. Many of the owners were empty-nesters who took the robot on as a member of the family. As mentioned earlier, the TES of being-with Aibo may or may not be like being with a dog, or it may be a bit like being with a baby. One of the owners says, “When I first got Aibo it was like having a new baby. It wasn't just a robot, because we had to raise it” (The New York Times, 2015). In terms of anthropomorphism, we might analyze this phenomenon as if the robot dog was replacing a biological dog, or a child no longer living at home. But neither of these explanations captures the actual role of the robot dog, or captures the way the owners interacted with their Aibos (shown on an untranscribed video). To the owners, their feelings of co-presence associated with their prior experiences of dogs or babies only brought them so far in attempting to capture their understanding of Aibo as an other. Here, the owners' second person perspective was reshaped given the asymmetry of the process at hand (see Figure 1 again). They implicitly and explicitly realized the fact that Aibo had capacities different from (but including some) of those of another peer, a dog, or a baby. Aibo had capacities of its own, and the owners treated it as a social interactor in its own right. The Aibo-owners' expectations and interactions with their robot dogs are a bit like the inverse of Darling and Turkle's interactions described above, at least at first glance. But in reality, the expectations of anthropomorphism aren't enough to capture the actual interactions between human and robot-as-social actor that we see in each case. Aibo's owners are already engaged in sociomorphing, but without the conceptual framework and language to describe it. Aibo clearly lacks autonomy in the enactive sense, but as new technologies emerge that might properly engage in social interactions with humans, we need a finer distinction in how we understand and experience sociality. And while there are no robots autonomous in the enactive sense (yet), perhaps gradations of autonomy are already emerging (the dog-like robots at Boston Dynamics described by Darling above may be an interesting candidate) and this framework guides us toward more productive design of these systems.

## Discussion

By combining the approaches of both PSM and sociomorphing, we have a new way to empirically study and theoretically examine artificial systems as they exist in interaction with us without constantly encountering epistemological roadblocks of systems built to *appear* social. In fact, Seibt and her collaborators have offered an in-depth ontology alongside the sociomorphing framework that attempts to provide descriptive tools related to different levels of simulation and asymmetry, carving up the problem space into at least five different kinds of simulation (Seibt, 2017; Seibt et al., 2020a). They have also created a new instrument meant to take the theoretical structures into the experimental sciences (Damholdt et al., 2020). Both traditional HRI research, as well as AGI work, are in need of this new terminology and framework, especially in light of the level of description PSM provides in studying social cognition. For example, in terms of Lying Cat, sociomorphing introduces a finer-grained approach to analyzing the actual systems at hand. In other words, we don't live in a universe with talking cats who have access to metaphysical truths, so if I interact with an actual cat who seems to be uttering “lying,” the framework of sociomorphing that I use appropriately leaves out linguistic capacities from the analysis. In the universe of *Saga*, that would be different. In the case of an actual dog vs. Aibo, anthropomorphism relies on language that may incorrectly equate the two, which doesn't allow for the literal ascription of social capacities onto either. Sociomorphing allows for us to take each of those systems as genuinely social, without only projecting human capacities onto them. In fact, if we take up sociomorphing broadly, other asymmetrical social interactions we regularly engage in (such as when we say hi to the neighbor's dog or while watching birds out the window) would be reframed as well, and we would have language and conceptual systems that accurately capture the real cognitive and emotional states of those systems. If each form of sociomorphing manifests in a TES, and TES is fundamentally tied to phenomenological experiences of co-presence as well as perspectival shifts, then AGI needs a reckoning with the overall framework used to analyze cognition broadly, but more specifically social cognition, most productively understood as PSM.

## Data Availability Statement

The original contributions presented in the study are included in the article/supplementary material, further inquiries can be directed to the corresponding author/s.

## Author Contributions

All authors listed have made a substantial, direct, and intellectual contribution to the work and approved it for publication.

## Conflict of Interest

The authors declare that the research was conducted in the absence of any commercial or financial relationships that could be construed as a potential conflict of interest.

## Publisher's Note

All claims expressed in this article are solely those of the authors and do not necessarily represent those of their affiliated organizations, or those of the publisher, the editors and the reviewers. Any product that may be evaluated in this article, or claim that may be made by its manufacturer, is not guaranteed or endorsed by the publisher.

## Footnotes

^1^We use AGI here in the more traditional sense of the term, as it overlaps with concepts of machine consciousness, machine mindedness, and early understandings of AI in general as an instantiation not merely of human-level intelligence, but of the concomitant mind as what is doing the thinking (Newell and Simon, 1961; Fuchs, 2021).

^2^We have in mind here Di Paolo et al.'s provocative assertion that, “Interactions are social as long as the autonomy of the agents is not dissolved… We do not restrict social interaction to the human species. As long as the terms of the definition can be verified, they can apply to cross-species interactions or interactions with robots that are autonomous in the sense intended” (2010, 443).

^3^An important note: In more than one place (2017, 2018), Seibt has laid out features of an entire ontology that tries to make sense of what she sometimes calls “simulated social interaction.” This ontology, called OASIS (Ontology of Asymmetric Social Interactions) lays out a number of specific features of these interactions, classifying different kinds of human-robot coordinated and collaborative actions through different sorts of simulations. This ontology can be closely tied to different kinds of sociomorphing, but the specific levels of her ontology don't quite capture our use of her concept. This is likely because we are attempting to expand this idea beyond the framework of social robotics into AGI/humanoid robotics work, which hopes to escape the focus on simulation and eventually emerge as simply doing.

^4^Coeckelbergh, as one example, states “I argue that designers have a responsibility for designing the role and narrative related to their artifact, and indeed designing the performance, but that the performance and the narrative created are also the responsibility of the user since they also co-create it” (Coeckelbergh, 2018, 73). This kind of feedback loop results in complex perspective-taking that we seem able to perform and create easily, but the theorizing behind such complexity is not nearly as easy.
